# Supplementary material for: A Kinetic Approach to Oxygen Radical Absorbance Capacity (ORAC): Restoring Order to the Antioxidant Activity of Hydroxycinnamic Acids and Fruit Juices
Source: Antioxidants (Basel). 2024 Feb 9;13(2):222. doi: 10.3390/antiox13020222 (PMC10886186; doi:10.3390/antiox13020222)
Supplement: Supplementary file 1 [file antioxidants-13-00222-s001.zip › antioxidants-2792815-supplementary.pdf]

Supplementary information

Table S1. Copasi: a Tutorial

<table>
<tr>
<td>Download the free software COPASI 4.41 (Build 283)</td>
<td>This is a simulator for biochemical networks. It is a joint project by the Hoops group (Biocomplexity Institute of Virginia Tech), the Mendes group (UCONN School of Medicine), the Kummer, and Sahle groups (University of Heidelberg). It works under the Artistic License 2.0 Copyright (c) 2000-2006, The Perl Foundation. You can download for free the software at the following website (10.12.2023): https://copasi.org/Download/
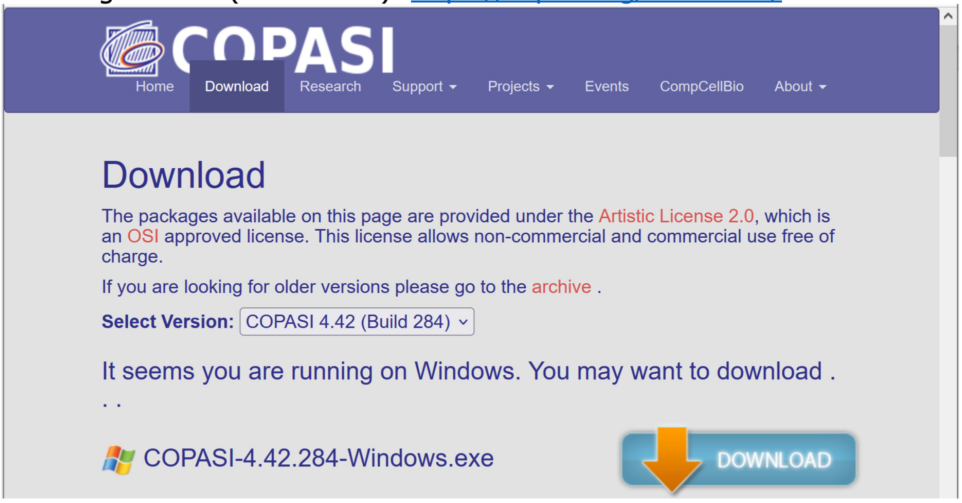

Different Versions running under Windows, Linux, Mac X operating systems are also freely available.</td>
</tr>
<tr>
<td>Initialize the Model</td>
<td>Once you open the COPASI software, you start with the model window. Here you can define preliminary information of the model, such as Model's name and units. Units are those internationally recognized, such as meter for length, second for time, mole for amount of substance, and liter for capacity.
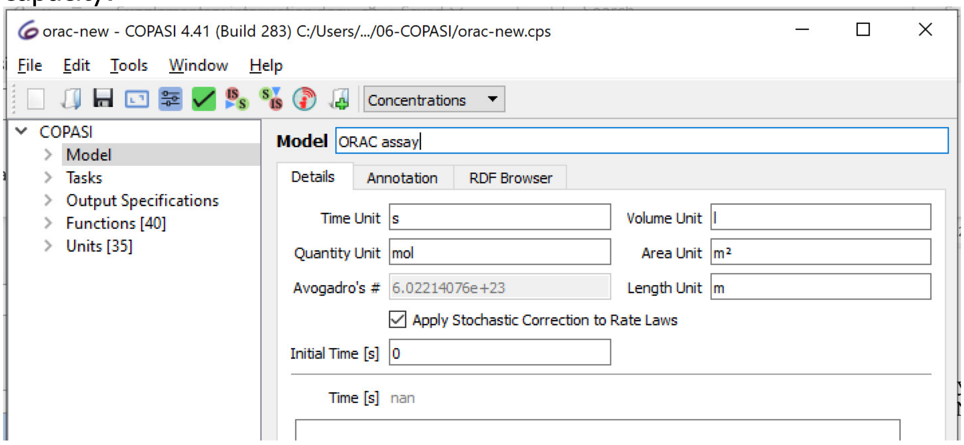
</td>
</tr>
</table>

<table>
<tr>
<td>Define a Reaction Mechanism</td>
<td>Clicking on the down arrow behind “Model”, a series of subfolders are accessible. The first to look for is the “Reaction” subfolder. Here you can define the mechanism of the kinetic model. For the ORAC assay, just copy the content in Table 1. Alternatively, simply import the SBML file (File / Import SBML…), which is also included in this work as Supplementary information. This file contains an exact copy of the information reported in the Figure below.

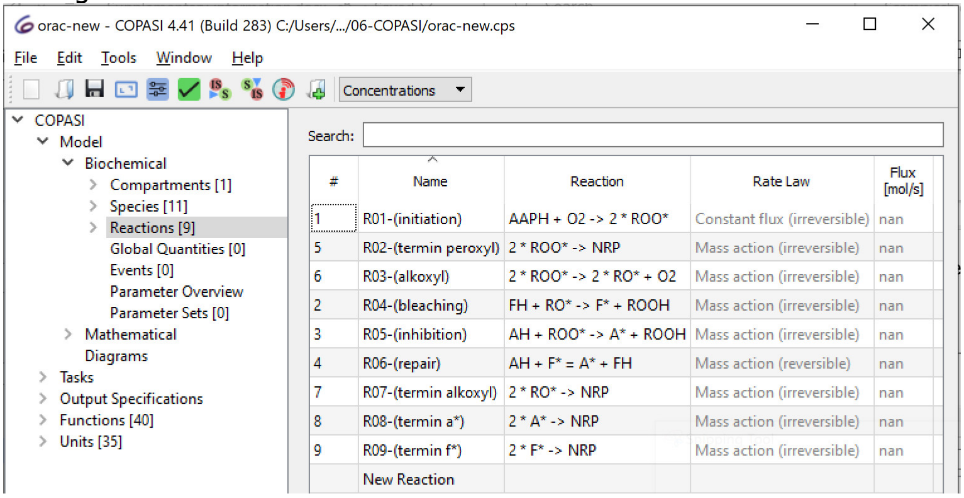

</td>
</tr>
<tr>
<td>Set the “initial guesses” for the kinetic parameters</td>
<td>For each reaction defined in the model, you must set a “known” or “guessed” value of each kinetic parameter that characterize the reaction. Fill all the rate constants as defined in Table 1 of the manuscript. For instance, for the initiation reaction R01, you should insert the rate value of the reaction following a rate law based on a “constant flux (irreversible)”. This is advantageous because we do not need to define a rate constant, which is not known, but we simply measure the rate, which can be measured or derived from the fitting routine. We also assume that the rate of free radicals generation is constant during the all experiment. Setting this rate value can be done by clicking on the “Reaction” menu, choosing the item with the name of the reaction that you want to modify, and finally, insert the rate value in the corresponding text box, as shown in the Figure below:

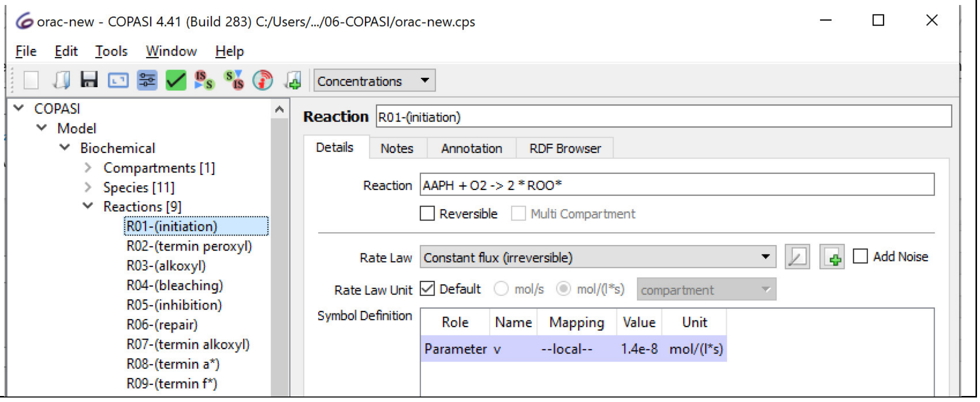

</td>
</tr>
</table>

<table>
<tr>
<td></td>
<td>
For setting the rate parameters of all the other reactions, the procedure is similar. However, this time you should select the rate law "mass action (irreversible)", like in the Figure below:

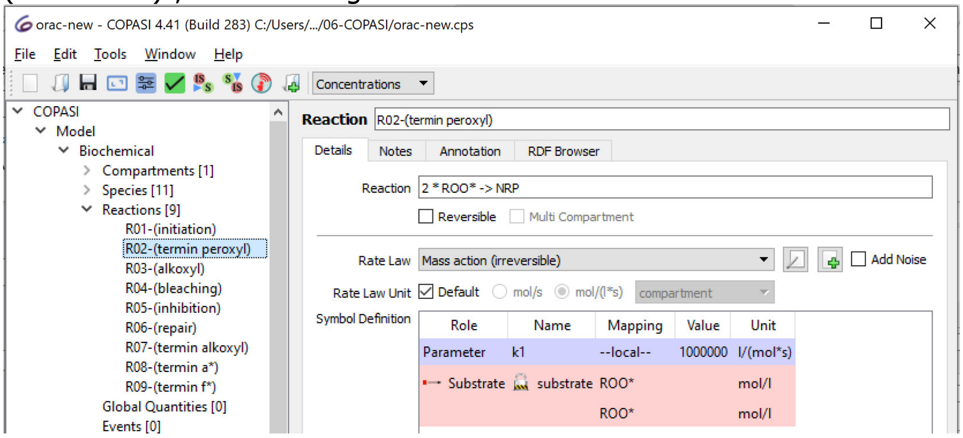

For the repair mechanism (R07), the reaction is an equilibrium. In this case, the reversed reaction must be set to 1×106 (Ms)-1, while the forward can be set to any value as this will be optimized later by COPASI during the fitting procedure. However, for performing a simulation, the forward rate constant must be also defined. Here, a guessed value of 8×106 (Ms)-1 was used.

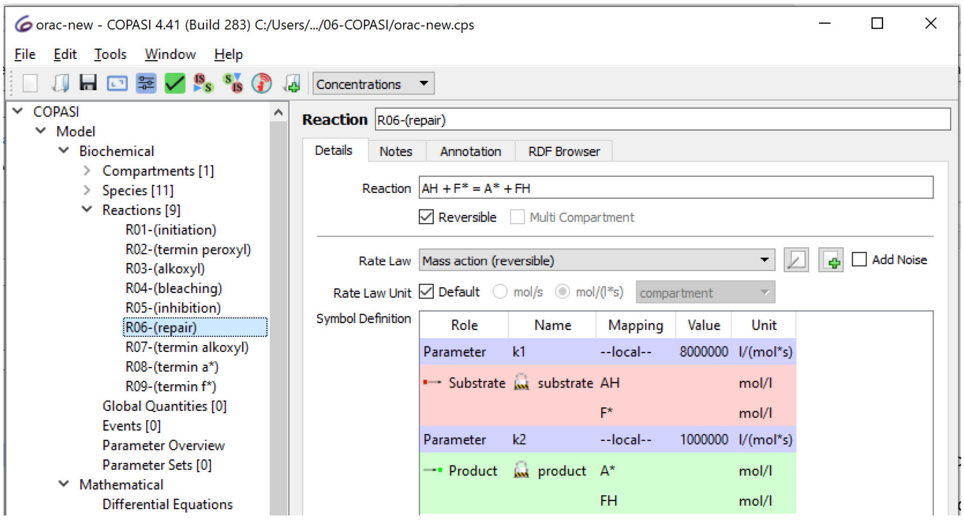

</td>
</tr>
<tr>
<td>Set the initial concentrations of reactants and products</td>
<td>Once the reactions are defined, the folder "Species" become populated by the chemical species identified by the names reported in the "Reaction" folder. Next step is to define the initial concentrations of reactants and products. Figure below shows the initial setup for the ORAC assay.</td>
</tr>
</table>

<table>
<tr><td></td><td>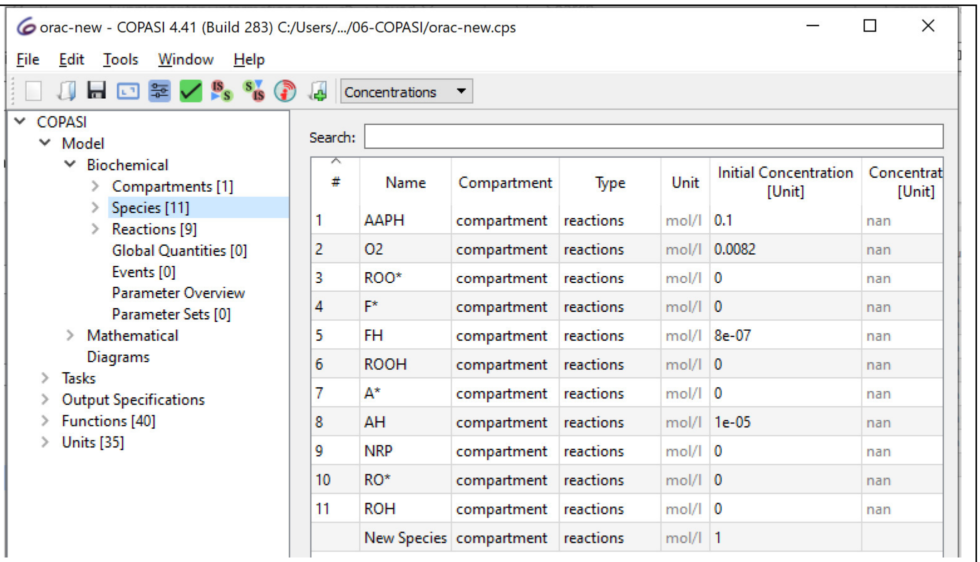

As you see, only four chemical species have an initial concentration that is different from zero. These species are the AAPH (0.1 M), oxygen (0.0082 M, obtained from n/V = p/RT, where p = 0.21, R = 0.082 L atm (K mol)-1, and T = 313 K), FH (8×10-7 M), and AH (varying from 1×10-5 M to 1×10-4 M, depending on the concentration and stoichiometry used in the experiment. Here, [AH] is set to 1×10-5 M). All the other species are set to zero.</td></tr>
<tr><td>Check the System of Differential Equations</td><td>COPASI generates a system of ordinary differential equations (ODE) based on conservation of mass balance. Each ODE represents the rate of one chemical species. The system of ODE used for the ORAC assay is presented below.</td></tr>
</table>

	$\frac{d([AAPH]\cdot V_{compartment})}{dt} = -V_{compartment}\cdot\text{"Constant flux (irreversible)"}(v_{\text{("R01-(initiation)")}})$<br>$\frac{d([O2]\cdot V_{compartment})}{dt} = -V_{compartment}\cdot\text{"Constant flux (irreversible)"}(v_{\text{("R01-(initiation)")}}) + V_{compartment}\cdot k1_{\text{("R03-(alkoxyl)")}}\cdot[\text{"ROO*"}]\cdot[\text{"ROO*"}]$<br>$\frac{d([\text{"ROO*"}]\cdot V_{compartment})}{dt} = +2\cdot V_{compartment}\cdot\text{"Constant flux (irreversible)"}(v_{\text{("R01-(initiation)")}}) - V_{compartment}\cdot k1_{\text{("R05-(inhibition)")}}\cdot[AH]\cdot[\text{"ROO*"}] - 2\cdot V_{compartment}\cdot k1_{\text{("R02-(termin peroxyl)")}}\cdot[\text{"ROO*"}]\cdot[\text{"ROO*"}] - 2\cdot V_{compartment}\cdot k1_{\text{("R03-(alkoxyl)")}}\cdot[\text{"ROO*"}]\cdot[\text{"ROO*"}]$<br>$\frac{d([\text{"F*"}]\cdot V_{compartment})}{dt} = +V_{compartment}\cdot k1_{\text{("R04-(bleaching)")}}\cdot[FH]\cdot[\text{"RO*"}] - V_{compartment}\cdot(k1_{\text{("R06-(repair)")}}\cdot[AH]\cdot[\text{"F*"}] - k2_{\text{("R06-(repair)")}}\cdot[\text{"A*"}]\cdot[FH]) - 2\cdot V_{compartment}\cdot k1_{\text{("R09-(termin f*)")}}\cdot[\text{"F*"}]\cdot[\text{"F*"}]$<br>$\frac{d([FH]\cdot V_{compartment})}{dt} = -V_{compartment}\cdot k1_{\text{("R04-(bleaching)")}}\cdot[FH]\cdot[\text{"RO*"}] + V_{compartment}\cdot(k1_{\text{("R06-(repair)")}}\cdot[AH]\cdot[\text{"F*"}] - k2_{\text{("R06-(repair)")}}\cdot[\text{"A*"}]\cdot[FH])$<br>$\frac{d([ROOH]\cdot V_{compartment})}{dt} = +V_{compartment}\cdot k1_{\text{("R04-(bleaching)")}}\cdot[FH]\cdot[\text{"RO*"}] + V_{compartment}\cdot k1_{\text{("R05-(inhibition)")}}\cdot[AH]\cdot[\text{"ROO*"}]$<br>$\frac{d([\text{"A*"}]\cdot V_{compartment})}{dt} = +V_{compartment}\cdot k1_{\text{("R05-(inhibition)")}}\cdot[AH]\cdot[\text{"ROO*"}] + V_{compartment}\cdot(k1_{\text{("R06-(repair)")}}\cdot[AH]\cdot[\text{"F*"}] - k2_{\text{("R06-(repair)")}}\cdot[\text{"A*"}]\cdot[FH]) - 2\cdot V_{compartment}\cdot k1_{\text{("R08-(termin a*)")}}\cdot[\text{"A*"}]\cdot[\text{"A*"}]$<br>$\frac{d([AH]\cdot V_{compartment})}{dt} = -V_{compartment}\cdot k1_{\text{("R05-(inhibition)")}}\cdot[AH]\cdot[\text{"ROO*"}] - V_{compartment}\cdot(k1_{\text{("R06-(repair)")}}\cdot[AH]\cdot[\text{"F*"}] - k2_{\text{("R06-(repair)")}}\cdot[\text{"A*"}]\cdot[FH])$<br>$\frac{d([NRP]\cdot V_{compartment})}{dt} = +V_{compartment}\cdot k1_{\text{("R02-(termin peroxyl)")}}\cdot[\text{"ROO*"}]\cdot[\text{"ROO*"}] + V_{compartment}\cdot k1_{\text{("R07-(termin alkoxyl)")}}\cdot[\text{"RO*"}]\cdot[\text{"RO*"}] + V_{compartment}\cdot k1_{\text{("R08-(termin a*)")}}\cdot[\text{"A*"}]\cdot[\text{"A*"}] + V_{compartment}\cdot k1_{\text{("R09-(termin f*)")}}\cdot[\text{"F*"}]\cdot[\text{"F*"}]$<br>$\frac{d([\text{"RO*"}]\cdot V_{compartment})}{dt} = -V_{compartment}\cdot k1_{\text{("R04-(bleaching)")}}\cdot[FH]\cdot[\text{"RO*"}] + 2\cdot V_{compartment}\cdot k1_{\text{("R03-(alkoxyl)")}}\cdot[\text{"ROO*"}]\cdot[\text{"ROO*"}] - 2\cdot V_{compartment}\cdot k1_{\text{("R07-(termin alkoxyl)")}}\cdot[\text{"RO*"}]\cdot[\text{"RO*"}]$
Start the simulation	COPASI allows to simulate the concentration evolution of each chemical species of the model by solving numerically the system of ODEs shown

	before. This is simply achieved by a user-friendly routing called "Time-Course", which is located within the folder "Taks". Once you have selected the folder Taks / Time Course, you can define the duration of the simulation (2000 s), the number of points that are simulated (2000 points). Ba default, the simulation routine uses LSODA and LSODAR routines from ODEPACK, a C algorithm that is particularly powerful to handle even stiff equations.

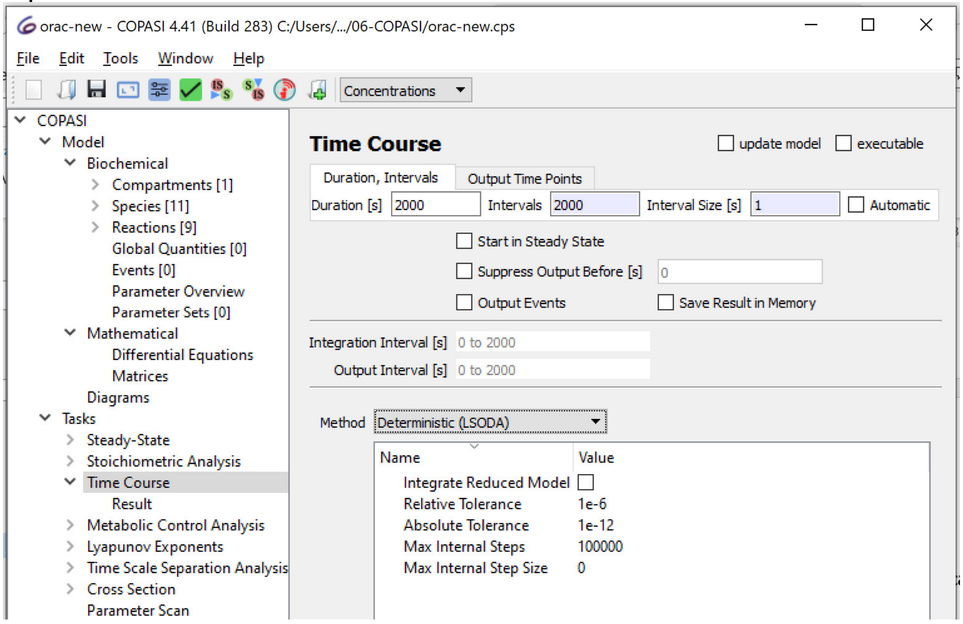

Before running the simulation, you have to choose one of the default plots available in COPASI by clicking the button "Output Assistant", as shown in the Figure below:

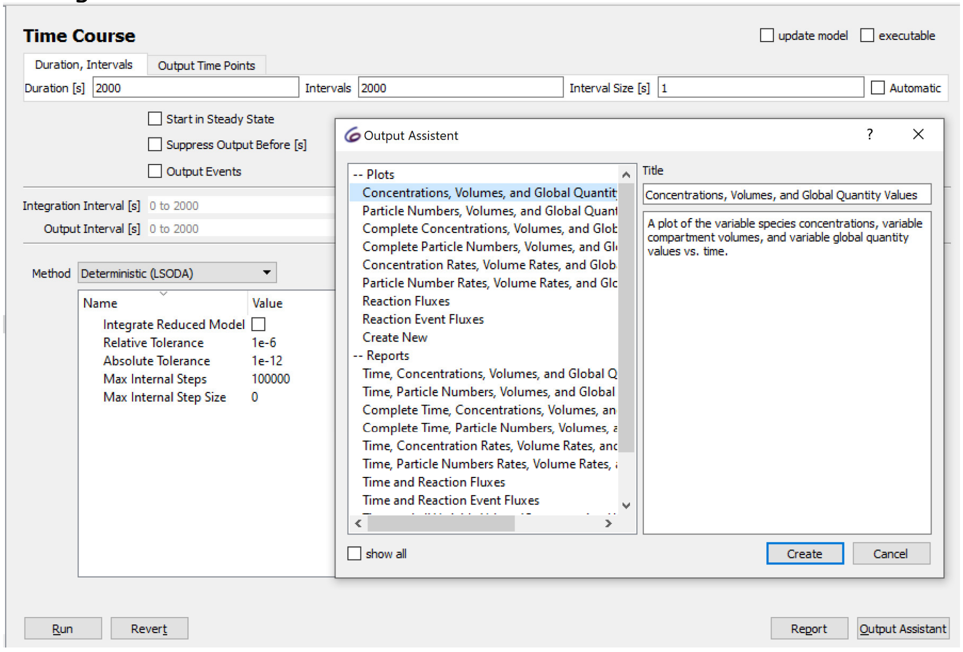

Figure S1. Screenshot of the Output Assistant routine of the COPASI Software to create plots

	For simulating the ORAC assay, it is sufficient to select the first plot available and click on "Create" button.<br>Now, executing the simulation by clicking on the button "Run", it is possible to visualize the result of the simulation, as shown in the following Figure:<br>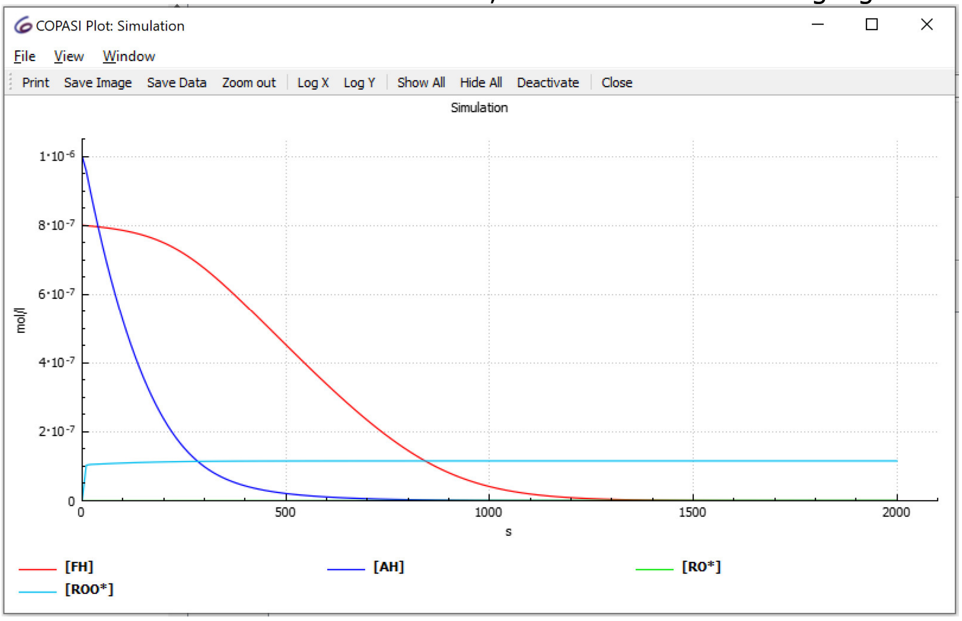<br><br>The Figure shows the transient changes of fluorescein (red line), the consumption of the antioxidant (blue line, [AH]0 = 1×10-6 M, with k5 set to 5×104 (Ms)-1. Also shown the production of peroxyl radicals (cyan line), while the alkoxyl radical formation is very small and not visible with the axis ranges of this plot.
Fit the experimental data	The values of the kinetic parameters can be optimized automatically using a fitting routine in COPASI. This routine is available under the "Task" folder, selecting the item "Parameter Estimation", as shown in the Figure below.

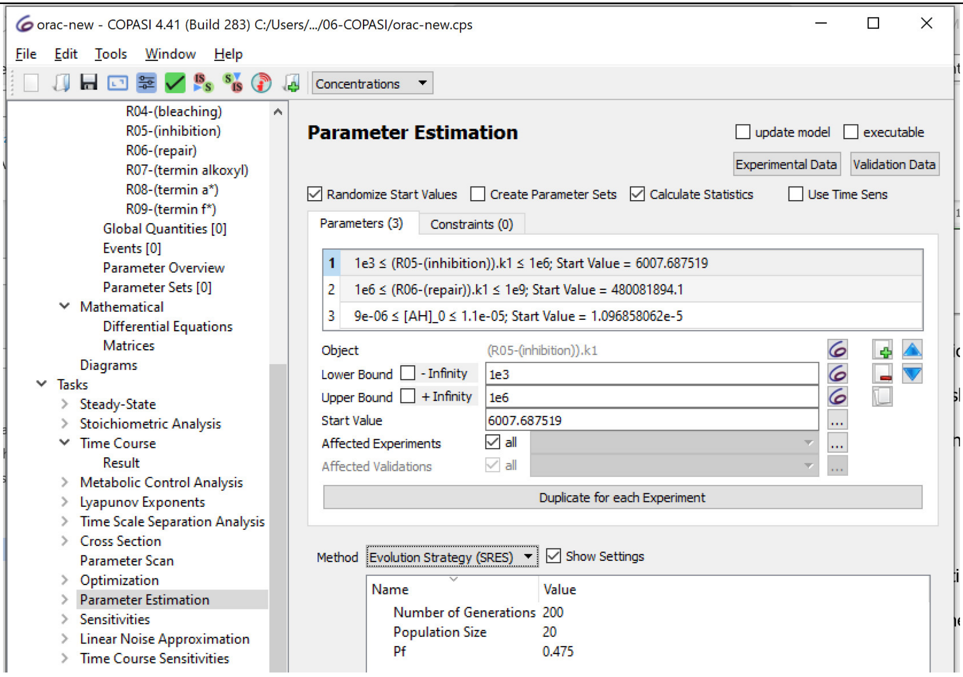

Within Parameter Estimation routine, you must choose the parameters to be optimized. In this example, three parameters were selected, respectively, the rate constant for the Reactions R05 and R06, and the initial concentration of the antioxidant [AH]0.

For each parameter, you can define the lower bounds within which the iterative fitting program in COPASI is allowed to find the optimal value that best match the experimental curve. A good practice is to flag the option "Randomize Start Value". This option allows COPASI to randomly set an initial guess value for each of the chosen parameters. This option is important for establishing the convergence of the optimal values of the parameters even if the initial value is changing. As method for finding the optimal parameters, we choose the SRES method, as shown in the Figure above. Details of this method are given in the manuscript.

Finally, the experimental data must be loaded by clicking on the button "Experimental Data". Experimental dataset must be saved in a .txt or .csv file, in a format like that represented in the following Figure:

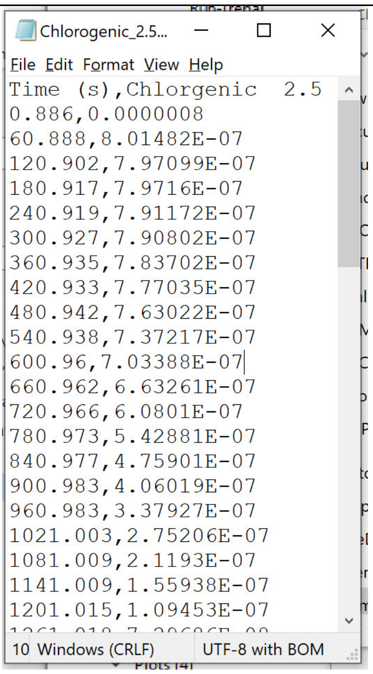

Next, the dataset can be loaded in COPASI by clicking on the button "Experimental Data" and than selecting the stored .csv. or .txt file, as it is shown in the following Fugure:

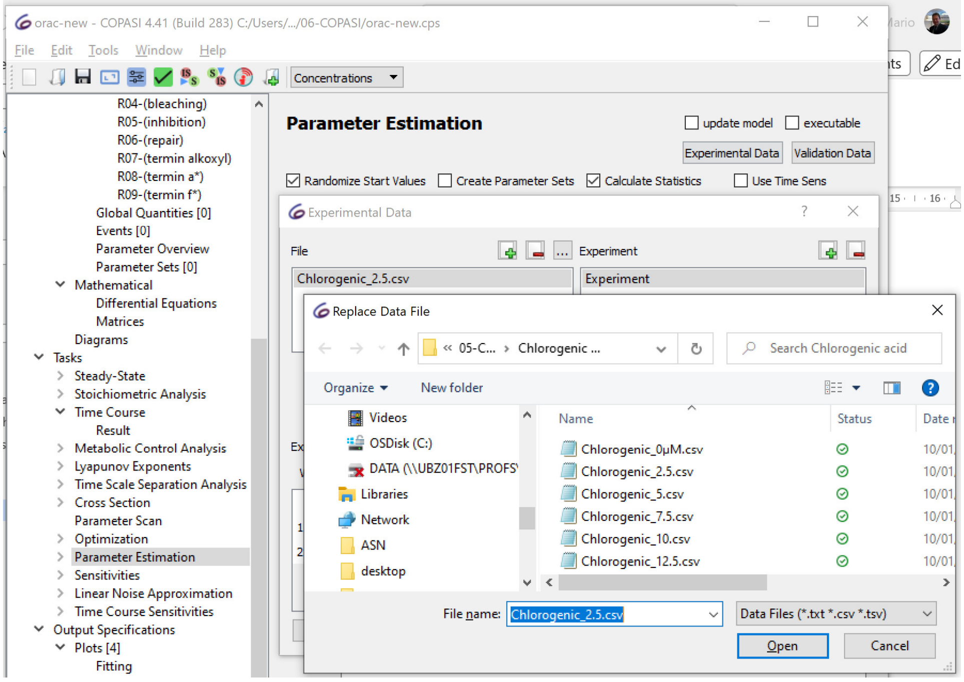

Once load the file, COPASI recognize automatically the structure of the dataset. However, you can define manually the first raw where the data start (i.e., "1"), the last row (i.e., "50"), and the separator symbol (i.e., ","). Also, be careful to select the flag "Time Course", which indicates that your file has in the first column a "time" variable.

Finally, associate column names. The first column, as said before, is automatically associated with the type "Time". The other column is FH, that must be associated with the corresponding chemical species defined in the model.

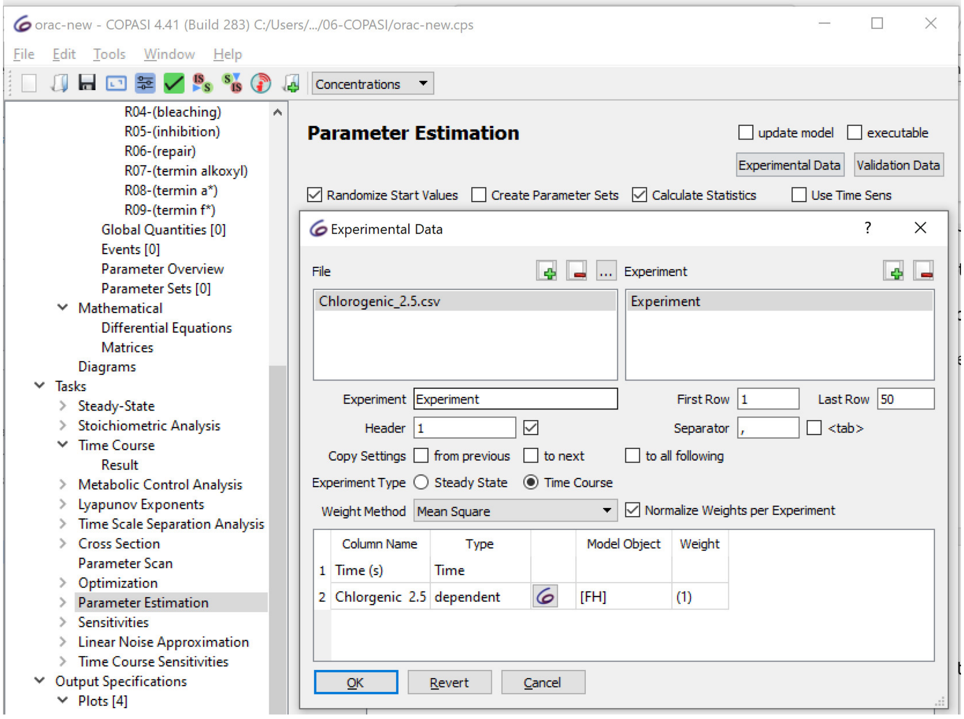

Finally press "OK", and, then, when returned to the main COPASI window, press the button "Run". The iterative fitting routine starts automatically finding the optimized values for the kinetic parameters of the chosen variables. A dialogue window appears, indicating the iterations that the fitting program is running and the goodness of the fit, indicated by the textbox "Best value", as shown in the following Figure:

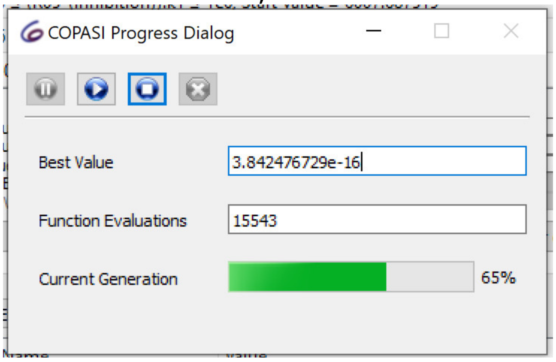

When the oterative fitting program has found the optimal value of the kinetic parameters that minimize the sum of squared difference between the experimental and simulated data, then the software provide a complete statistical analysis of the fitting routine, as shown in the Figure below:

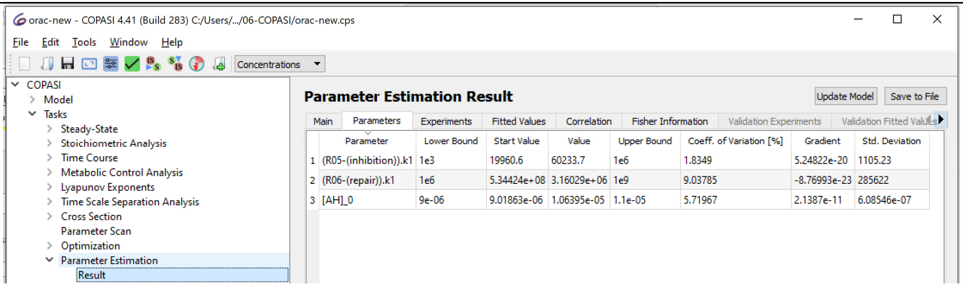

Also, by creating the plot with the "Output Assistant" button, it is possible to create a plot that shows the experimental data (as points) and the simulated data (as line), as shown in the following Figure:

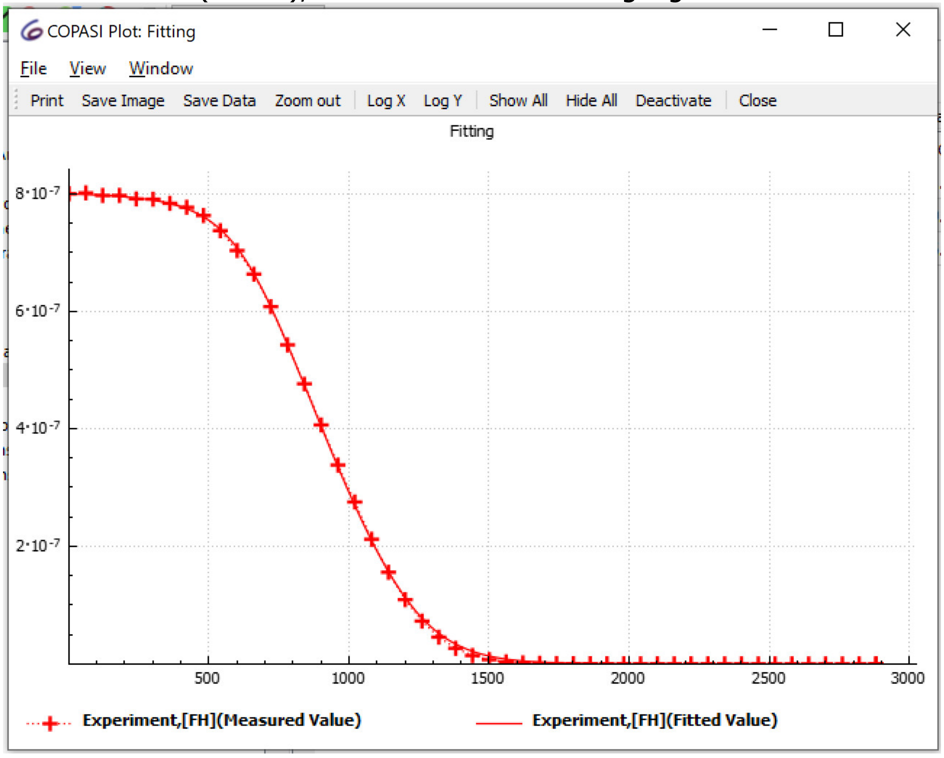

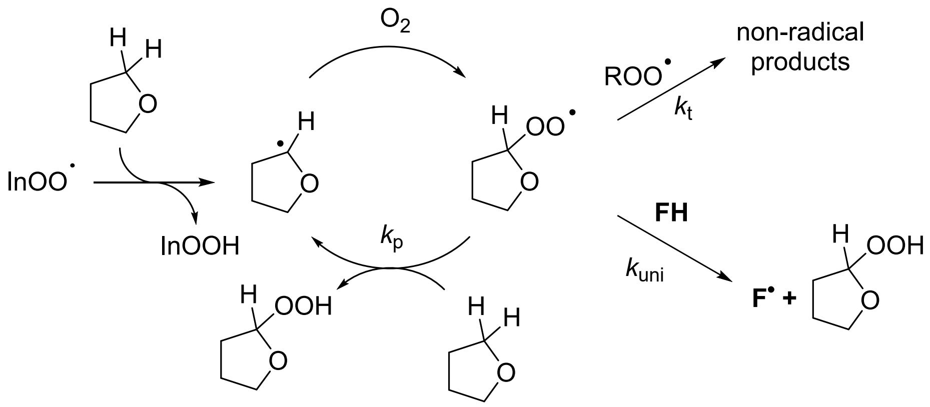

Scheme S1. Mechanism of autoxidation of tetrahydrofuran (THF) initiated by InOO• radicals in the presence of fluorescein (FH).

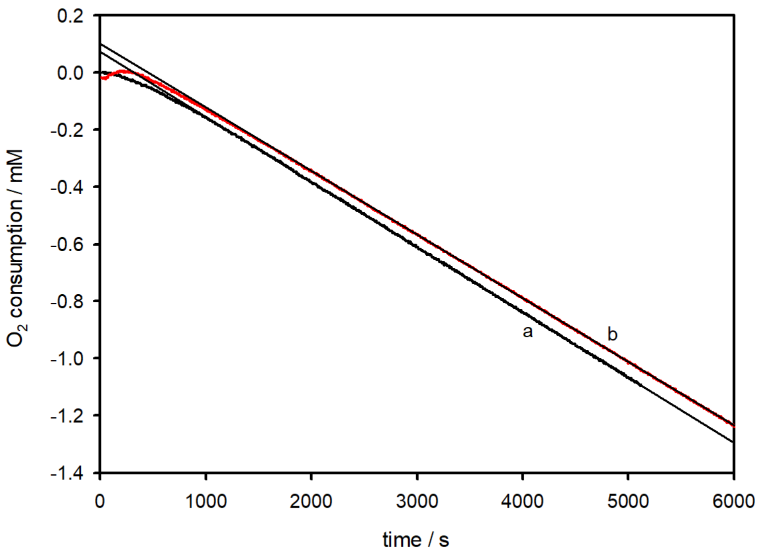

Figure S1. O2 consumption during the autoxidation of tetrahydrofuran (3.1 M) in water at pH 7.0, initiated by AAPH (50 mM) at 30°C, in the absence (a) and in the presence of fluorescein 25 μM. Oximetry Analysis of Fluorescein Reactivity Toward Peroxyl Radicals. Autoxidation were performed in a two-channel oxygen uptake apparatus, based on a Validyne DP 15 differential pressure transducer built in our laboratory. The peroxyl radical-trapping activity was evaluated by studying the inhibition of the thermally initiated autoxidation of tetrahydrofuran (THF) in buffered water at pH 7.4. THF has been purified by distillation before the experiment to remove the inhibitor. In a typical experiment, THF (0.5 mL), AAPH and 100 mM phosphate buffer were added to a round bottomed flask to reach an overall volume of 4 mL. The sample was equilibrated with an identical reference solution containing an excess of Trolox. The oxygen consumption in the sample was measured after calibration of the apparatus from the differential pressure recorded with time between the two channels. The rate constant of the reaction between alkylperoxyl radicals (ROO•) and fluorescein in water was obtained

from the slopes of O2 consumption in the presence and in the absence of fluorescein (FH) (R_{in} and R_0, respectively) by equation 1, where R_i is the initiation rate (1.5×10^{-8} M s^{-1}), *n* is stoichiometric coefficient of FH and $2k_t$ is the termination constant of THF (6.6×10^7 $M^{-1}s^{-1}$) [49]. The experiments were performed in triplicate.

$$(R_0/R_{in}) / (R_{in}/R_0) = (n\ k_{uni}\ [FH]) / (R_i\ 2k_t)^{1/2} \quad (1)$$
